# Prevalence of Alopecia Areata in Saudi Arabia: Cross-Sectional Descriptive Study

**DOI:** 10.7759/cureus.10347

**Published:** 2020-09-10

**Authors:** Abdulmajed Al-ajlan, Mahmmed E. Alqahtani, Sami Alsuwaidan, Abdulaziz Alsalhi

**Affiliations:** 1 Dermatology, King Saud Bin Abdulaziz University for Health Sciences College of Medicine, Riyadh, SAU; 2 Dermatology, National Guard Hospital, King Abdullah International Medical Research Center, Riyadh, SAU; 3 Dermatology, King Saud University Medical City, Riyadh, SAU

**Keywords:** alopecia areata, hair, alopecia, hair loss, hair fall

## Abstract

Background

Alopecia areata (AA) is a common autoimmune disorder worldwide that affects the hair. Population differences have been observed in disease prevalence and clinical features, but no studies have examined AA prevalence at a large scale. In Saudi Arabia, information is lacking about AA characteristics.

Objectives

A quantitative, observational, cross-sectional study was conducted to assess AA prevalence, characteristics, and gender differences in Saudi Arabia.

Materials and methods

The study has used a validated Arabic questionnaire that targeted Saudi Arabia residents with a history of AA.

A validated Arabic questionnaire was used to target Saudi Arabia residents with a history of AA, and the data collection instrument and written informed consent were distributed on Twitter and Facebook after permission from the Institutional Review Board. Prior to the study, accuracy validation for correct diagnosis by participants was performed in a 50-volunteer pilot test, which indicated an acceptable level of 96% accuracy. The questionnaire included high-quality images of different AA types and the collected data focused on variables such as the age of onset, affected body parts, treatment type, and family history of AA.

Results

A total of 5,362 participants returned completed questionnaires, of whom 741 (13.8%) had experienced AA at least once in their lives. Most were aged 11-30 years (69%), and the mean age of diagnosis was 18.6 years. Thirty-six percent (36%) of those with AA reported having a first-degree relative with the disease, and cross-sectional prevalence was 5.2%. Three-hundred fifty-nine (359; 18.9%) males and 382 (11%) females had AA. Twenty-nine point four percent (29.4%) of AA patients recovered in less than three months of AA onset, and 59.4% recovered in less than one year.

Conclusion

AA prevalence in Saudi Arabia is higher than in Western countries with a lower age of onset than in the former. AA affected males more than females and the mean age of onset was lower in the latter. Both male gender and young age of onset implied a worse prognosis.

## Introduction

Alopecia areata (AA) is an inflammatory autoimmune disease characterized by non-scarring hair loss in circular-to-oval areas, which is mostly reversible [1-2]. The disease varies in terms of its severity, from small, simple, and well-circumscribed areas of hair loss to severe and progressive total hair loss [3]. AA is usually diagnosed based on clinical presentation, but skin biopsies may be needed in certain situations [2]. The management of AA patients is challenging because there is no cure or proven effective therapy for the disorder [4]. It is a relatively common disease with a lifetime incidence of approximately 2%, but first-degree relatives of AA patients often have a higher lifetime risk of 24% [5-7]. AA is the most prevalent autoimmune disease, and health-related quality of life appears to be poor in AA patients, especially when it leads to severe disfigurement and psychosocial distress [5,8]. Despite the high incidence and significance of this disorder, no previous studies have been conducted to determine AA prevalence in Saudi Arabia. Therefore, drawing on a sample of social media users living in Saudi Arabia, this study’s goal was to identify and evaluate the prevalence, patient characteristics, and currently used treatment methods for AA in Saudi Arabia.

## Materials and methods

A quantitative, observational, cross-sectional study was undertaken using an online Arabic-language questionnaire created with Google Forms after permission from the Institutional Review Board (IRB). The questionnaire and written informed consent was distributed on Twitter and Facebook between May 2019 and September 2019, and people living in Saudi Arabia were targeted. The first part of the questionnaire focused on demographic data, including age, gender, and country of residence. The second part focused on AA-related information, including the age of onset, affected body part, treatment type, and AA family history. Participants younger than 15 years old, if they reported suffering from AA, were told to seek help from their parents. The required sample size to achieve a 95% confidence interval was 4,706, and a precision level of 0.004 was chosen based on the worldwide calculated lifetime incidence of 2% for AA [5]. Data were analyzed using version 21 of the Statistical Package for the Social Sciences (SPSS) software (IBM Corp., Armonk, NY).

## Results

Participants

After excluding 239 participants who had completed questionnaires but who were not Saudi Arabia residents, the total number of online questionnaire respondents was 5,359. Of the participants, 64.6% were female and 35.4% were male as shown in Table 1. Table 2 shows the age of the study population.

**Table 1:** Population - Gender

Gender	Number	Percent
Female	3462	64.6%
Male	1897	35.4%

**Table 2:** Study Population Age Most participants were aged between one and 30 years (86.2%).

Age	Number	Percent
1 – 10 Years	23	0.4%
11 – 20 Years	2393	44.7%
21 – 30 Years	2204	41.1%
31 – 40 Years	460	8.6%
41 – 50 Years	197	3.7%
>51	82	1.5%
Total	5359	100%

Prevalence

The number of participants who reported having AA, either previously or at the time of the survey, was 741 (13.8%). Two-hundred eighty (280; 5.2%) were suffering from active lesions. AA prevalence in males was 359 out of 1,897 (18.9%) while it was 382 out of 3,462 (11%) in females, as shown in Table 3.

**Table 3:** Diseased Population Age

Age	Number	Percent
1 – 10 Years	161	21.7%
11 – 20 Years	307	41.4%
21 – 30 Years	206	27.8%
31 – 40 Years	53	7.2%
41 – 50 Years	11	1.5%
>51	3	0.4%
Total	741	100%

As shown in Table 4, the age of onset was earlier in females when compared to males, at 16 and 22 years, respectively, while the average age of onset across the two genders was 19 years.

**Table 4:** Age at Time of Diagnosis in Years

Age (years)	Male (%)	Female (%)	Total (%)
1 – 10	33 (9.2)	128 (33.5)	161 (21.7)
11 – 20	135 (37.6)	172 (45)	307 (41.4)
21 – 30	147 (40.9)	59 (15.4)	206 (27.8)
31 – 40	36 (10.0)	17 (4.5)	53 (7.2)
41 – 50	6 (1.7)	5 (1.3)	11 (1.5)
51 <	2 (0.6)	1 (0.3)	3 (0.4)
Total	359 (100.0)	382 (100.0)	741 (100.0)

Family history

Based on the collected data, 36.4% of the affected 741 participants had a first-degree relative with AA. There was a slight male gender predominance (37.5% males and 35.3% females).

Body site

For all participants other than males older than 20 years, the scalp was the most affected area; otherwise, the beard was the most common. The age range in which most male participants experienced scalp AA was 11-20 (44.2%) while beard AA occurred mainly in participants aged 21-30 (57.7%), as shown in Table 5.

**Table 5:** Body Part Affected by Alopecia Areata

Body part affected by alopecia areata	Male (%)	Female (%)	Total (%)
Scalp only	218 (60.6)	364 (95.3)	582 (78.4)
Beard only	129 (35.8)	1 (0.3)*	130 (17.5)
More than one area**	13 (3.6)	17 (4.5)	30 (4.0)
Total	360 (100.0)	382 (100.0)	742 (100.0)

Duration and treatment

AA duration seemed unrelated to treatment in this data set, but 29.4% of the participants experienced AA for three months. A clear female gender predominance was observed, with 22.6% males and 35.9% females (Table 6).

**Table 6:** Disease Duration

Duration (Months)	Male (%)	Female (%)	Total (%)
< 3	81 (22.6)	137 (35.9)	218 (29.4)
3 – 6	53 (14.8)	39 (10.2)	92 (12.4)
6 – 9	44 (2.3)	38 (9.9)	82 (11.1)
9 – 12	16 (4.5)	32 (8.4)	48 (6.5)
12 <	165 (46)	136 (35.6)	301 (40.6)
Total	359 (100.0)	382 (100.0)	741 (100.0)

Two-hundred eighty (280; 38%) participants reported suffering from AA at the time of completing the survey, which indicates a cross-sectional prevalence of 5.2%. Forty-five percent (45%) of scalp AA cases were resolved in less than six months while 32% of beard AA cases were resolved in this period. While there was a noticeable discrepancy in choice of treatment in the participants, herbal medicines were the most common (17.1%), followed by topical medications (14.8%), and local injections (12.4%). Eleven point six percent (11.6%) of the participants recovered spontaneously (Table 7).

**Table 7:** Kind of Treatment Used Post Recovery From Alopecia Areata * the rest still have active disease

Type of treatment used	No. of patients	Percent
Did not use anything	86	18.7%
Local injections	92	20%
Herbal remedies	127	27.5%
Topical medications	110	23.9%
Other	46	10%
Total	461 *	100.0%

## Discussion

No previous studies have examined the epidemiology of AA in Saudi Arabia, but the literature is rich in research projects indicating that AA is a common disease worldwide with varying prevalence rates and early age of onset [9-14]. AA prevalence in a multicenter hospital-based study in Japan was 2.5% while our study reported a prevalence rate of 5.25% [9]. This high figure could be explained by the elevated rate of consanguinity in Saudi Arabia. Additionally, in our study, 36.4% of AA patients had a first-degree relative with AA. In the literature, several studies have assessed the gender distribution of AA patients, and conflicting results have been reported. Guzmán-Sánchez et al. [15] and Tan et al. [16] reported a female predominance while Sharma et al. [11] and Kavak et al. [17], consistent with our study, identified a male predominance.

This study’s results indicate that members of the Saudi population tend to develop AA at an earlier age than their counterparts in other countries. For example, in our study, individuals aged 11-20 were the most affected while Furue et al. [9] identified individuals aged 31-40 in Japan. A study in West Los Angeles, California, involved 800 AA patients, and it reported that 48% developed AA before 20 years of age [13]. In contrast, 63% of our study population developed AA by 20 years of age, and only 9% after 30 years. There is a noticeable difference in the mean age of diagnosis between the Olmsted population and our study’s population. For males, the Olmsted population’s mean age of diagnosis was 32 years for males, and 36 years for females, which contrasts with our population’s male and female ages of 16 and 22, respectively [10]. Our results indicate that AA onset was earlier in females, that better prognosis is linked to the female gender, and younger age of onset was associated with a worse prognosis. Specifically, disease duration was less than three months in 22.6% and 35.6% of males and females, respectively, and more than one year in 46.0% and 35.6%. Similar to a clinical AA study conducted in Mexico, our study reported that the most common site of AA involvement in males was the scalp (60.7%) [15].

Treatment of AA depends on both the presentation and severity of the condition [18]. Most participants in this study experienced remission with herbal treatments (27.5%), but the possibility of spontaneous remission may have played a role. One way to account for the popularity of herbal medications in this research population is by referencing cultural factors. For example, in Saudi Arabia, it is commonly believed that herbal medications and plants such as garlic can have potent effects as medical treatments for various conditions. Other medical treatments include topical and local injections, both of which are linked to remission from the perspective of patients (Table 6).

Shellow et al.’s US-based study reported that 42% of AA patients had a family history of the condition [13]. A study in Kuwait also reported a strong family history of AA, with 51% of AA children (n = 215) having a positive family history [19]. However, none of the percentages given in these previous studies represents first-degree relatives only. In Yang et al.’s China-based study conducted between 2001 and 2003, the researchers distributed a questionnaire to AA patients (n = 1,032) at Anhui Medical University [20]. Eight point four percent (8.4%) of patients reported a positive family history of AA, with a prevalence of 1.6% among first-degree relatives.

Our study’s results confirm that family history is an important factor in AA. This is especially the case in view of the fact that 37% of participants with AA also had a first-degree relative with the condition. The study also revealed that younger age of onset and beard lesions were both associated with a worse prognosis and delays in remission. Fifty-nine percent (59%) of participants recovered from their condition within one year while 29% recovered in three months or less. Most scalp AA cases were resolved in less than three months, and most beard AA cases took up to six months to resolve. This study could be limited by the use of an online questionnaire, particularly in terms of the possibility of selection bias and systematic bias.

## Conclusions

AA prevalence in Saudi Arabia is higher than it is in Western countries, and the age of onset appears to be lower. In contrast to other autoimmune diseases, this study’s results indicate that AA affected males in our sample more than females and the mean age of onset was higher in the former. Furthermore, both male gender and young age of onset implied worse prognosis, and beard lesions were identified as lasting for a longer period of time when compared to scalp lesions. Thirty-six percent of those with previous experience of AA reported having a first-degree relative with the condition, which suggests that heritability plays a major role in AA pathophysiology. This study highlights the importance of further study in this area, especially because 5.2% of the participants were suffering from active lesions, 40% of them for more than one year.

## References

[1] Alzolibani A (2011). Epidemiologic and genetic characteristics of alopecia areata (part 1). Acta Dermatovenerol Alp Pannonica Adriat.

[2] Spano F, Donovan JC (2015). Alopecia areata. Part 1: pathogenesis, diagnosis, and prognosis. Can Fam Physician.

[3] Wasserman D, Guzman-Sanchez DA, Scott K, Mcmichael A (2007). Alopecia areata. Int J Dermatol.

[4] Hordinsky M, Junqueira AL (2015). Alopecia areata update. Semin Cutan Med Surg.

[5] Press D (2015). Epidemiology and burden of alopecia areata: a systematic review. Clin Cosmet Investig Dermatol.

[6] Wienker TF, Weert J De, Lambert J (2006). Familial aggregation of alopecia areata. J Am Acad Dermatol.

[7] Khoshdel A, Shekari A, Paydary K, Shekari I (2016). Alopecia areata: the role of stressful events and an estimate of lifetime risk in first-degree relatives. J Arch Mil Med.

[8] Shi J, Feng H, Lee J, Chen WN (2013). Comparative proteomics profile of lipid-cumulating oleaginous yeast: an iTRAQ-coupled 2-D LC-MS/MS analysis. PLoS One.

[9] Furue M, Yamazaki S, Jimbow K (2011). Prevalence of dermatological disorders in Japan. J Dermatol.

[10] Mirzoyev SA, Schrum AG, Davis MDP, Torgerson RR (2014). Lifetime incidence risk of alopecia areata estimated at 2.1 percent by Rochester Epidemiology Project, 1990-2009. J Investig Dermatol.

[11] Siiakma VK, Kumar B (1992). Profile of alopecia areata in Northern India. Int J Dermatol.

[12] Melton LJ, Muller SA, Suman VJ, Moshell AN, Melton LJ III (1995). Incidence of alopecia areata in Olmsted County. Mayo Clin Proc.

[13] Shellow WVR, Edwards JE, Koo JYM (1992). Profile of alopecia areata: a questionnaire analysis of patient and family. Int J Dermatol.

[14] Tan E, Tay Y, Giam Y (2002). A clinical study of childhood alopecia areata in Singapore. Pediatr Dermatol.

[15] Guzmán‐Sánchez DA, Villanueva‐Quintero GD, Alfaro NA, McMichael A (2007). A clinical study of alopecia areata in Mexico. Int J Dermatol.

[16] Tan E, Tay Y, Goh C, Giam YC (2002). The pattern and profile of alopecia areata in Singapore - a study of 219 Asians. Int J Dermatol.

[17] Kavak A, Ye NÍ, Parlak AH, Gökdemir G, Anul H, Baykal C (2008). Alopecia areata in Turkey: demographic and clinical features. J Eur Acad Dermatol Venereol.

[18] Messenger AG, Mckillop J, Farrant P, McDonagh AJ, Sladden M (2012). British Association of Dermatologists guidelines for the management of alopecia areata 2012. Br J Dermatol.

[19] Nanda A, Al‐Fouzan AS, Al‐Hasawi F (2002). Alopecia areata in children: a clinical profile. Pediatr Dermatol.

[20] Yang S, Yang J, Liu JB (2004). The genetic epidemiology of alopecia areata in China. Br J Dermatol.

